# High-quality, customizable heuristics for RNA 3D structure alignment

**DOI:** 10.1093/bioinformatics/btad315

**Published:** 2023-05-11

**Authors:** Michal Zurkowski, Maciej Antczak, Marta Szachniuk

**Affiliations:** Institute of Computing Science, Poznan University of Technology, 60-965 Poznan, Poland; Institute of Computing Science, Poznan University of Technology, 60-965 Poznan, Poland; Department of Structural Bioinformatics, Institute of Bioorganic Chemistry, Polish Academy of Sciences, 61-704 Poznan, Poland; Institute of Computing Science, Poznan University of Technology, 60-965 Poznan, Poland; Department of Structural Bioinformatics, Institute of Bioorganic Chemistry, Polish Academy of Sciences, 61-704 Poznan, Poland

## Abstract

**Motivation:**

Tertiary structure alignment is one of the main challenges in the computer-aided comparative study of molecular structures. Its aim is to optimally overlay the 3D shapes of two or more molecules in space to find the correspondence between their nucleotides. Alignment is the starting point for most algorithms that assess structural similarity or find common substructures. Thus, it has applications in solving a variety of bioinformatics problems, e.g. in the search for structural patterns, structure clustering, identifying structural redundancy, and evaluating the prediction accuracy of 3D models. To date, several tools have been developed to align 3D structures of RNA. However, most of them are not applicable to arbitrarily large structures and do not allow users to parameterize the optimization algorithm.

**Results:**

We present two customizable heuristics for flexible alignment of 3D RNA structures, geometric search (GEOS), and genetic algorithm (GENS). They work in sequence-dependent/independent mode and find the suboptimal alignment of expected quality (below a predefined RMSD threshold). We compare their performance with those of state-of-the-art methods for aligning RNA structures. We show the results of quantitative and qualitative tests run for all of these algorithms on benchmark sets of RNA structures.

**Availability and implementation:**

Source codes for both heuristics are hosted at https://github.com/RNApolis/rnahugs.

## 1 Introduction

Comparing the 3D structures of RNA molecules is one of the important problems in computational biology and bioinformatics. Comparative analysis of polymer folds is based primarily on structural alignment, and followed by hunting for similarities between data objects given as clouds of atoms. It applies to establish homology between molecules following their 3D conformations (Dietmann and Holm 2001; Blazewicz et al. 2005), discover conserved 3D structure motifs (Miskiewicz et al. 2017; Valdes-Jimenez et al. 2019), identify tertiary structure families and perform structure-based classifications (Lo Conte et al. 2000), assess the quality of algorithms that predict 3D models of molecules (Lukasiak et al. 2015; Gong et al. 2019), create non-redundant sets for benchmarking and structural studies (Leontis and Zirbel 2012; Adamczyk et al. 2022), etc. Structural alignment consists in such an arrangement of one structure vs. the other in a 3D space that the average distance between the corresponding atoms is as small as possible. If the arrangement depends on the sequence, that is, optimizes the distance between the corresponding nucleotides of two molecules having the same sequences, we call it superimposition. Otherwise, when the matching is sequence-independent, we perform structural alignment. Structures can be aligned in a rigid or flexible way. The former involves a rigid transformation (rotations and translations) of an entire structure that is superimposed onto the other. Unfortunately, it has the disadvantage of leaving entire regions without alignment, even if they are similar and could superpose very well locally. To overcome these cons, aligners of the new generation usually apply flexible alignment. It consists of building an alignment through a sequence of local transformations focused on fragments of structures. If alignment aims to find local similarities and solve the problem of maximum common substructures, the result should include the location and length of the matched, implicitly similar fragments and their RMSD, root mean square deviation (Kabsch 1978). The latter measures the quality of alignment (Maiorov and Crippen 1994; Lukasiak et al. 2013). Otherwise, aligners can be also applied to assess a global similarity of the structures. Therefore, when selecting the alignment algorithm, one should take into account the problem to be solved—whether the chains compared have the same length, whether we know their sequences and if they are similar, whether the alignment is dependent or independent on the sequence, and whether we need rigid or flexible alignment. All of this impacts the alignment optimization procedure, including the objective function and computational complexity.

To date, several algorithms have challenged the problem of aligning 3D RNA structures. They include ARTS (Dror et al. 2005), SARA (Capriotti and Marti-Renom 2008), LaJolla (Bauer et al. 2009), R3D Align (Rahrig et al. 2010), iPARTS (Wang et al. 2010), SETTER (Hoksza and Svozil 2012), STAR3D (Ge and Zhang 2015), SupeRNAlign (Piatkowski et al. 2017), Rclick (Nguyen et al. 2017), RMalign (Zheng et al. 2019), and RNA-align (Gong et al. 2019). SARA and iPARTS are no longer available. ARTS is the only program in the pool that implements rigid superposition; all the others apply flexible alignment. ARTS depends on the commercial DSRR software (Lu and Olson 2003), which makes it inaccessible to many users. LaJolla generates sparse output (only a PDB file with transformed coordinates of structures, but no indication of which nucleotides were aligned), which limits its usefulness and disables benchmarking. Other algorithms produce results with a wide range of quality. Most perform quite well when aligning structures that show high similarity, and noticeably worse when dealing with homologously distant RNAs. Each follows a different scheme and relies on different initial assumptions; for example, some work in sequence-dependent and independent modes, others only in one of them; a few accept the RMSD threshold at the input, while most do not; some allow processing large datasets as they are available as standalone applications; the other do not, etc. All of them use coarse-grained models to align, but the grain differs between methods. Table 1 selects the important features of these programs.

**Table 1.** Selected features of available algorithms to align 3D RNA structures.

	Application type	Input format	RMSD threshold	Processing mode		Coarse-grained model	Alignment based on	Multiple alignments	Alignment-related output data		
				seq-dep	seq-indep				Aligned nts	Actual RMSD	3D alignment
LaJolla	Standalone	PDB			✓	2 angles/3nts	Torsion angles				✓
R3D Align	Webserver, standalone	PDB			✓	4-nt neighborhoods	3D coordinates		✓		✓
SETTER	Webserver	PDB	✓		✓	1 bead (P)	2D units		✓	✓	✓
STAR3D	Standalone	PDB	✓		✓	1 bead (pseudoatom)	2D and 3D geometry		✓	✓	✓
SupeRNAlign	Webserver, standalone	PDB			✓	4-nt neighborhoods	3D coordinates		✓		✓
Rclick	Webserver	PDB, mmCIF	✓		✓	1 bead (C3’)	3D coordinates	✓	✓	✓	✓
RMalign	Standalone	PDB			✓	1 bead (C3’)	2D and 3D geometry		✓	✓	✓
RNA-align	Webserver, standalone	PDB, mmCIF		✓	✓	1 bead (C3’)	2D and 3D geometry		✓	✓	✓
GEOS (seq-dep)	Standalone	PDB, mmCIF	✓	✓	✓	3 beads (pseudoatoms)	3D coordinates		✓	✓	✓
GEOS (seq-indep)	Standalone	PDB, mmCIF	✓	✓	✓	3 beads (pseudoatoms)	3D coordinates		✓	✓	✓
GENS (seq-dep)	Standalone	PDB, mmCIF	✓	✓	✓	3 beads (pseudoatoms)	3D coordinates	✓	✓	✓	✓
GENS (seq-indep)	Standalone	PDB, mmCIF	✓	✓	✓	3 beads (pseudoatoms)	3D coordinates	✓	✓	✓	✓

In this work, we introduce geometric search (GEOS) and genetic algorithm (GENS), two novel algorithms to solve the flexible alignment problem of 3D RNA structures. GEOS is a dedicated geometry-oriented heuristics; GENS applies a genetic search approach. Both algorithms work in two modes, sequence-dependent and sequence-independent. They are complementary in applications. GENS performs better for similar structures and, therefore, is more useful in the alignment of distant homologs or various models of the same structure. GEOS is better for structures that differ significantly, so we recommend it for the problem of finding maximal common substructures. GEOS returns one solution; GENS can find multiple alignments of similar quality if they exist. Both allow users to define the maximum RMSD of the alignment to be found. The effectiveness of GEOS and GENS was confirmed in tests on a set of more than 1000 RNA structures. In this article, we show the results of these computational experiments and compare our algorithms with other available methods that address the problem of aligning RNA tertiary structures.

## 2 Materials and methods

Let *M* denote the 3D RNA model to align with the target structure *T* with an RMSD that does not exceed the threshold value *U*. Both GEOS and GENS operate on a coarse-grained representation of the tertiary structure of RNA. Therefore, their first step is data preprocessing, which involves the transformation of *M* and *T* from a full atom to a 3-bead coarse-grained model. In the latter case, each nucleotide is represented by three pseudoatoms: one for the phosphate group, one for the ribose group, and one for the nitrogenous base. The spatial coordinates of the pseudoatom determine the geometric center in the set of corresponding atoms.

Both methods return the longest alignment found within the given RMSD threshold *U*. If more alignments of the same length satisfy the threshold, the one with the lowest actual RMSD is returned.

### 2.1 Geometric search (GEOS)

The Geometric Search algorithm follows a three-step procedure. In the first step, it finds promising alignment kernels; next, it expands kernel-based alignments and compares them to select the best (cf. Supplementary Fig. S1). The best solution is the longest alignment with RMSD≤U. By default *U *=* *3.5 Å but users can select a different value between 0 and 20Å and specify it as input parameter. The default value has been chosen based on the experiences of CASP and RNA-Puzzles where structures with RMSD≤3.5 Å are considered similar (Antczak et al. 2016).

*Identification of kernels*: Kernel *K* should be made up of three pairs of well-aligned nucleotides, *K*=$\{N_{TA}$–$N_{MA}$, $N_{TB}$–$N_{MB}$, $N_{TC}$–$N_{MC}\}$; rmsd(*K*) ≪U. In each pair, one nucleotide belongs to the target *T* and its partner to the model *M*; $N_{TA}$, $N_{TB}$, $N_{TC}\;\in T$; $N_{MA}$, $N_{MB}$, $N_{MC}\;\in M$. Nucleotides of the kernel that are members of the same structure do not have to be adjacent to each other in the polymer chain. Moreover, a single nucleotide can belong to more than one kernel. Each promising kernel has a high probability of being part of an optimal solution.

A single kernel is searched as follows. Two nucleotides, $N_{TA}$ and $N_{TB}$, are drawn in the target structure *T*. Then, any two nucleotides, $N_{MA}$ and $N_{MB}$, are drawn in model *M* and optimally aligned with $N_{TA}$ and $N_{TB}$. The algorithm checks whether rmsd($N_{TA}$–$N_{MA}$, $N_{TB}$–$N_{MB}$)$<U_{2}$; $U_{2}$ is the RMSD threshold defined for two pairs of nucleotides, $U_{2}<U$, by default $U_{2}$=0.65 Å. If not, it rejects $N_{MA}$ and $N_{MB}$ and continues to draw nucleotides in the model until it finds those that fit the RMSD threshold $U_{2}$. GEOS then completes the kernel by adding the third pair. It takes a random nucleotide $N_{TC}$ from the target, $N_{TC}\notin \{N_{TA}$, $N_{TB}\}$ and a random nucleotide $N_{MC}$ from the model, $N_{MC}\notin \{N_{MA}$, $N_{MB}\}$, and adds them to the kernel. Next, it checks whether rmsd($N_{TA}$–$N_{MA}$, $N_{TB}$–$N_{MB}$, $N_{TC}$–$N_{MC}$)$<U_{3}$; $U_{3}$ is the RMSD threshold for three pairs of nucleotides, $U_{2}<U_{3}<U$, by default $U_{3}$=1.0 Å. If the inequality is not satisfied, GEOS continues to draw the third nucleotide in the model. If it cannot find such a nucleotide in *M*, it discards the third target nucleotide from the kernel, takes the other random $N_{TC}$ from *T*, and starts drawing its partner from the model again. Following this scheme, GEOS creates many independent kernels, $K_{1}$, $K_{2}$, $K_{3}$…, which are passed to the second stage. The algorithm then builds structural alignments operating on these kernels and selects the best as a result of the computation.

*Building kernel-based alignments*: The search for alignment proceeds independently for each kernel found in the previous step. Kernel $K_{i}$ initiates the creation of a structural alignment $L_{i}$ between the target *T* and the model *M*. The procedure is as follows. The $K_{i}$ kernel is added to the alignment $L_{i}$. The entire structure of the model is transformed (translated and rotated) to align with the target. GEOS performs rigid body alignment using the rotation and translation matrices calculated for the kernel. This means that the transformation of *M* aims to minimize RMSD only between the nucleotides that make up the kernel. Next, $L_{i}$ is extended by a new pair of nucleotides $N_{TD}$–$N_{MD}$. This is the pair with the smallest Euclidean distance in the set of all non-$L_{i}$ pairs. The algorithm computes RMSD of current alignment, rmsd(*Li*). As long as rmsd(*Li*)<U and there are still non-$L_{i}$ nucleotides, the algorithm continues to add nucleotide pairs to $L_{i}$ and recalculates the RMSD of the current solution. GEOS builds multiple independent alignments in parallel, compares them, and selects the best. The best solution is the longest alignment found in all threads. If more alignments have the same length, the one with the lowest RMSD is kept. If multiple solutions have the same length and RMSD score, the first one is returned. It works until it meets one of the stopping criteria (cf. Section 2.3).

### 2.2 Genetic search (GENS)

Genetic algorithms (GA) are randomized optimization techniques guided by the principles of evolution, with the ability to implicitly parallelize (Booker et al. 1989). They operate in a search space containing chromosomes, that is, potential solutions to a problem encoded in a specific data structure. GA starts by creating a random initial population. Each individual (potential solution) in the population is evaluated using a fitness function. Selected ones go for crossover and mutation and—after application of these operators—yield a new generation. The algorithm then iterates the evaluation, selection, crossover, and mutation until the stop condition is met. The following paragraphs describe the parameters of GENS, the genetic algorithm dedicated to the 3D RNA alignment problem.

*Chromosome*: An individual is represented as a vector *V* of length *n*, where *n* is the number of nucleotides in the target structure *T*. If the *j*-th nucleotide of model *M* has been aligned with the *i*-th nucleotide of *T*, then *V*[*i*] = *j*; otherwise *V*[*i*] = 0.

*Initial population*: The population consists of c randomly generated individuals, by default c = 200. At first, each individual $I_{k}$ is represented by a vector $V_{k}$ = [0, 0, …, 0], *k *=* *1.*c*. Next, each vector is randomly filled with the indices of the unassigned nucleotides of *M* (all nucleotides in the model have the same probability of being selected). The continuity of the chain is preserved; adjacent indexes—except those of unaligned nucleotides—form a unique and monotonic sequence of consecutive numbers; in this sense, the vector [0,0,2,4,3,0] represents an inadmissible solution.

*Fitness function*: When evaluating individuals (i.e. structural alignments), the fitness function takes into account three criteria that describe the quality and length of the alignment. The algorithm seeks to minimize the root mean square deviation, maximize the number of aligned nucleotides, and minimize the number of incorrectly aligned nucleotides.

*Selection*: Fifteen percent of the fittest individuals in the current population are selected as the most promising seed for a new generation. They are subject to mutations (with probability *P *=* *.74), crossovers (with *P *=* *.25), and random seeding (with *P *=* *.01) to populate the new generation.

*Crossover*: Crossover is a probabilistic process that generates offspring chromosomes by exchanging information between parents. Two random individuals, $I_{i}$ and $I_{j}$ (parents), of the current population are crossed to create a new individual $I_{k}$ that is added to the population. Crossover involves drawing two numbers *x*, *y*; x<y and x,y∈<1, n>. The subvector $I_{i}[x..y]$ is copied to $I_{k}[x..y]$; the remaining cells of $I_{k}$ are filled with values taken from the corresponding cells in $I_{j}$. If $I_{k}$ contains two identical values, one (chosen at random) is converted to 0.

*Mutation*: Randomly selected individuals are subjected to single-point mutations (with 65% chance), double-point mutations (with 15% chance), triple-point or quadruple-point mutations (both with 10% chance). The following mutation types can be applied (with the same probability): (i) unassign previously assigned nucleotide of *T*; (ii) assign any available nucleotide of *M* to the unassigned nucleotide of *T*; (iii) assign any available nucleotide of *M* to the already assigned nucleotide of *T*; and (iv) swap assignments between two randomly selected nucleotides of *T*. Simple diagrams showing four mutation variants are presented in Supplementary Fig. S2.

### 2.3 Stopping criteria of GEOS and GENS

Both algorithms stop if one of the following criteria is satisfied: (i) all target residues have been aligned with a given RMSD threshold; (ii) processing time has reached the upper bound $c_{l}$ (by default $c_{l}$ = 300*s*); (iii) GENS only: the best current alignment has not been improved or refined for at least $c_{g}$ generations (by default $c_{g}$ = 300); and (iv) The best current alignment has not been improved or refined for the $b_{i}$ amount of time.

The size of the time buffer $b_{i}$ increases by time unit δt if the alignment improves, that is, its length increases or RMSD decreases. δt depends on several parameters. Parameter values can be set in the configuration file.

### 2.4 Implementation

GEOS and GENS are multi-threaded algorithms. By default, they compute by making use of all available CPU cores. The number of cores involved can be limited by the user, who can set the appropriate parameter value in the configuration file. The performance of both algorithms depends on a number of parameters. Some of them are input parameters; the others can be set via the configuration file. All parameters are described in the readme file available on GitHub (https://github.com/RNApolis/rnahugs). GEOS and GENS are single command-line applications. They are run with input data; the mandatory ones are two files in PDB/mmCIF format with 3D RNA structures to be aligned. Both algorithms were implemented in Java using the Maven package and tested with Java 11 and Maven 3.6.3.

## 3 Results

We verified the performance of GEOS and GENS and compared them with those of other algorithms that align 3D RNA structures. We conducted several computational experiments to examine various properties of the algorithms. In the first, quantitative (Section 3.1), we ran standalone apps in the sequence-independent mode for a benchmark set from RNA-Puzzles. In the second set of experiments (Section 3.2), we applied all the methods for selected 3D structures in sequence-dependent and sequence-independent modes. In the above experiments, we looked at the length of the alignments, their quality, the variety of solutions, and the ability of the algorithms to process structures of various sizes. Finally, we conducted experiments focusing exclusively on GEOS and GENS. We computed their execution times depending on the instance size and checked the repeatability of the results of both heuristics (Section 3.3).

### 3.1 Quantitative analysis of alignments

In this multi-model experiment, we used the benchmark set *S* (https://github.com/RNA-Puzzles/standardized_dataset) available within the RNA-Puzzles resources (Magnus et al. 2020). The collection contains standardized data for 1028 structures and is divided into 22 subsets, $S=\underset{i=1..22}{\cup }S_{i}$. Each of them corresponds to one RNA-Puzzles challenge and includes a reference structure $T_{i}$ and a set $M_{i}$ of models generated computationally by various tools, $M_{i}=\underset{j=1..k}{\cup }M_{ij}$, where $k=|S_{i}|-1$. The collection contains structures 41–188 nucleotides long. These data were parsed and preprocessed to match the requirements of third-party alignment programs. The changes consisted of renumbering models and atoms in multichain structures.

GEOS and GENS were compared with algorithms implemented as standalone applications that provide detailed output data on alignments, making them comparable. They include R3D Align (Rahrig et al. 2010), STAR3D (Ge and Zhang 2015), SupeRNAlign (Piatkowski et al. 2017), RMalign (Zheng et al. 2019), and RNA-align (Gong et al. 2019). Among them, only STAR3D allows users to define the maximum RMSD of the alignment to be searched for, although the algorithm occasionally returns solutions that exceed the threshold. GEOS and GENS use the RMSD threshold as a hard constraint. Thus, they return solutions of expected quality: fragments that match below the given threshold. In the experiment, we used this option to level the playing field for all the methods tested. We performed a comparative analysis based on the lengths of alignments of the same quality found by different algorithms. The procedure was run separately for each competitive algorithm $A_{k}\in \{$R3D Align, STAR3D, SupeRNAlign, RMalign, RNA-align} in the following way: (i) the algorithm $A_{k}$ was run for each model $M_{ij}\in S$ to align it with the corresponding target $T_{i}$; (ii) for each $M_{ij}$, we calculated $\mathrm{rms}d_{k}(M_{ij},T_{i})$, the RMSD of each alignment found by $A_{k}$; (iii) for each $M_{ij}\in S$, GEOS and GENS were run in sequence-independent mode with threshold *U* = $\mathrm{rms}d_{k}(M_{ij},T_{i})$; (iv) for each $M_{ij}$, we compared the lengths of alignments found by the three algorithms ($A_{k}$, GEOS and GENS); and (v) for each $M_{ij}$, we calculated the actual RMSD of the alignment found by GEOS and GENS. The solutions found for the models in the set *S* had quality in the various ranges; R3D Align: 0.65–59.56 Å, STAR3D: 1.22–8.52 Å; SupeRNAlign: 1.83–8.72 Å; RMAlign: 2.00–9.98 Å, and RNA-align: 2.00–10.92 Å (Table 2). RMalign and RNA-align use exactly the same algorithm for sequence-independent alignment, so they give similar results. For some models, three algorithms failed or aligned single nucleotides (<10% of the model). These cases (8 models for R3D Align, 7 for STAR3D, and 764 for SupeRNAlign) were classified as outliers and discarded from further study. Thus, the analysis was performed for the entire set (1028 structures) when comparing GEOS and GENS with RMalign and RNA-align, and for a subset including 1020/1021/264 models when comparing our algorithms with R3D Align/STAR3D/SupeRNAlign.

**Table 2.** Percentage of sequence-independent alignments with RMSD (Å) falling in defined ranges found for RNAs from the RNA-Puzzles set.

	Solutions with RMSD in the range				Unresolved cases
	[0, 3)	[3, 6)	[6, 10)	[10, ∞)	
R3D Align	7.0%	13.5%	24.1%	53.6%	0.8%
STAR3D	17.8%	81.3%	0.2%	—	0.7%
SupeRNAlign	2.9%	8.95%	12.8%	—	74.3%
RMalign	4.9%	85.0%	10.1%	—	—
RNA-align	5.8%	86.4%	7.7%	0.1%	—

**Figure 1.**
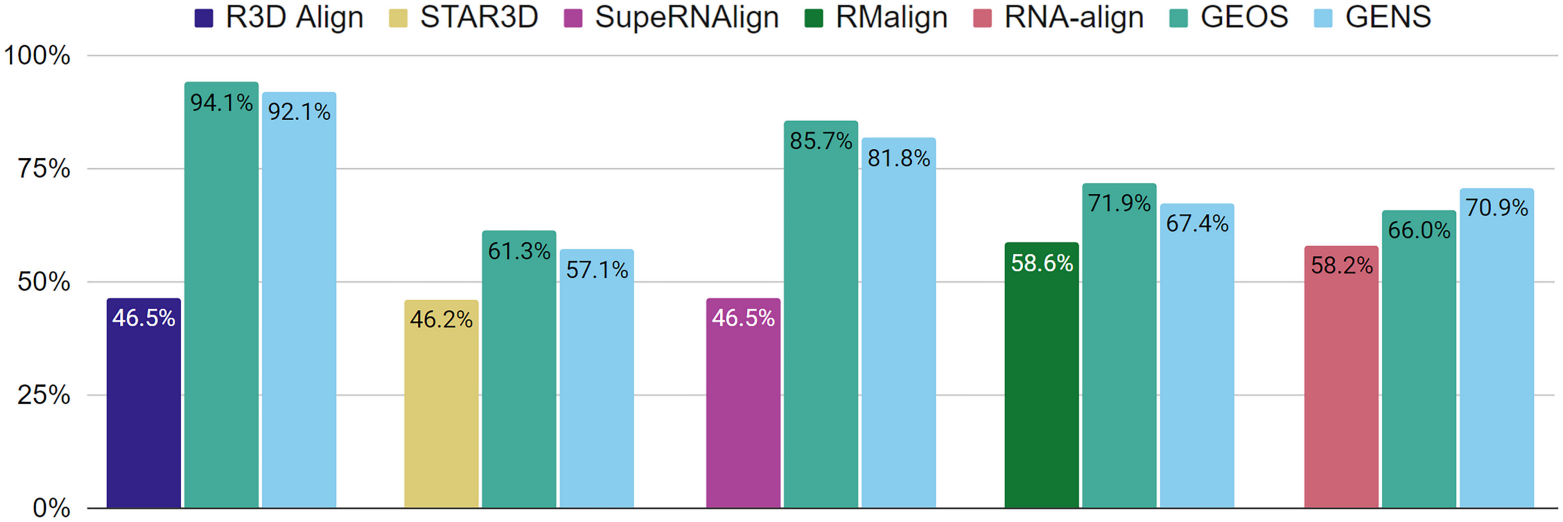
The coverage of target structures by sequence-independent alignments found by R3D Align, STAR3D, SupeRNAlign, RMalign, RNA-align, GEOS, and GENS for 3D RNA structures from the RNA-Puzzles set. The percentage for each algorithm was calculated for all aligned models from the RNA-Puzzles set.

With the resulting alignments, we focused on their lengths. First, we computed the percentage coverage of the targets by alignments (Fig. 1). Compared to the others, GEOS and GENS find noticeably longer alignments of the same quality. GEOS emerges as the winner here. Fragments found by this algorithm are, in total, twice as long as the solutions generated by R3D Align and 40% longer than those of SupeRNAlign. Among competing algorithms, RMalign and RNA-align work best. Their alignments are 8–13% shorter than those of GEOS and GENS. Supplementary Fig. S3 allows us to analyze the alignments from the perspective of each subset $S_{i}$ (each challenge of the RNA-Puzzles) separately. It shows the percentage of each target structure $T_{i}$ covered by the respective fragments aligned by each algorithm. The coverage was calculated as the average in the set of all models in $M_{i}$. In the case of SupeRNAlign, no data are shown for some puzzles because the algorithm did not find solutions there.

For each model $M_{i}\in S$, we checked which algorithm found the longest alignment. We made a pairwise comparison; the algorithm that aligned a longer fragment than its competitor was credited with winning the duel and the other with a loss. We counted the number of times each algorithm won or lost the duel. The aggregate results showing the performance of GEOS and GENS versus the others are shown in Table 3, details in Supplementary Table S1. GEOS won the most duels (90.55%) and lost the least (4.08%). GENS scored 86.97% of wins and 10.52% of losses. Furthermore, GEOS and GENS have fought each other 4361 times. In these skirmishes, 2679 wins (61.43%) went to GEOS and 480 (11%) to GENS.

**Table 3.** GEOS and GENS against other algorithms.

	Duels held	Duels won		Duels lost	
	#	#	%	#	%
R3D Align	2040	6	0.29	2024	99.21
STAR3D	2042	102	4.99	1862	91.21
SupeRNAlign	528	1	0.18	522	98.86
RMalign	2056	259	12.59	1613	78.45
RNA-align	2056	269	13.08	1718	83.56
GEOS	4361	3949	90.55	178	4.08
GENS	4361	3793	86.97	459	10.52

### 3.2 Example alignments of RNA 3D structures

**Figure 2.**
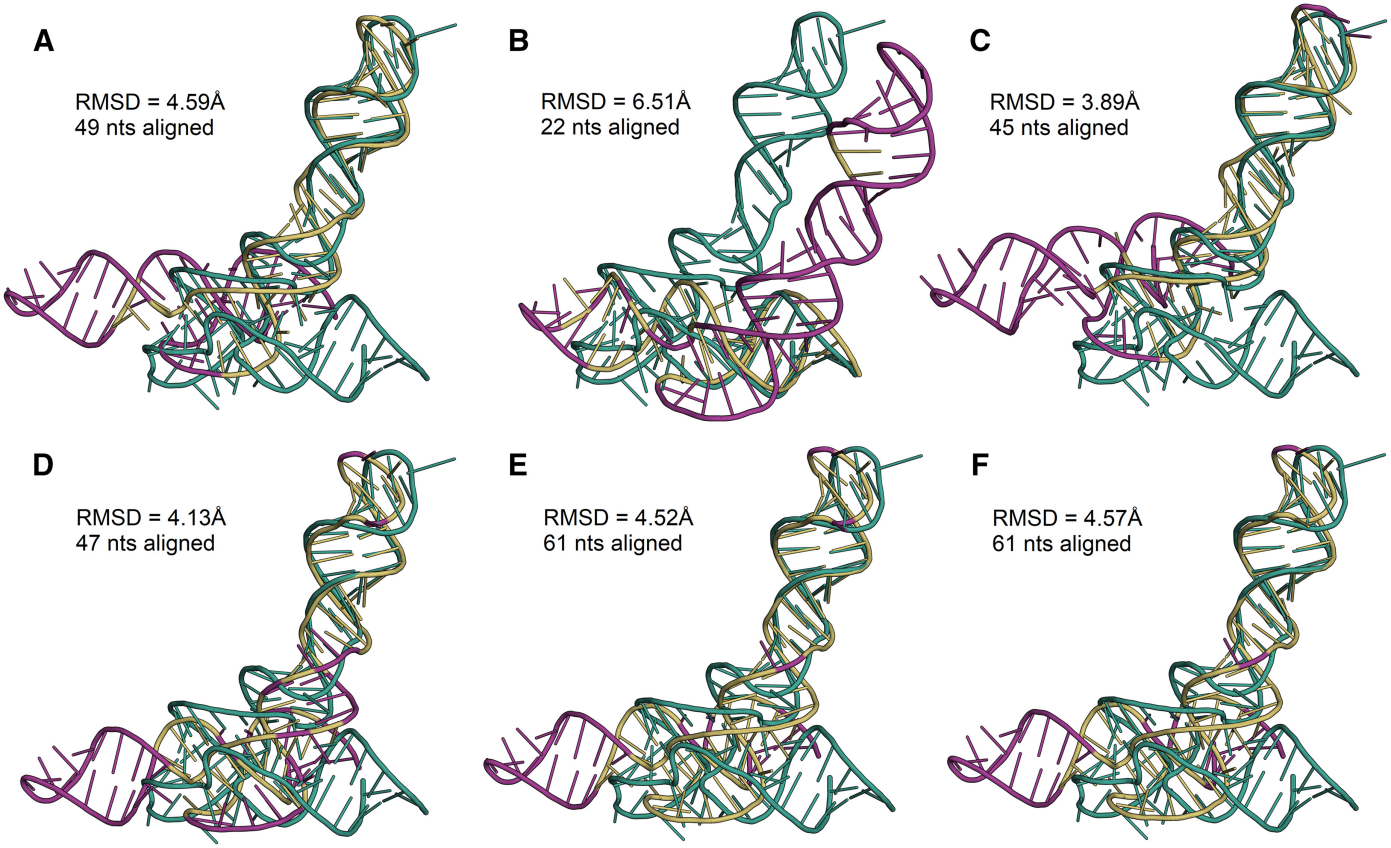
Sequence-independent alignment of the PZ3-Das-2 model with Puzzle 3 target found by (A) RMalign, (B) SETTER, (C) STAR3D, (D) Rclick, (E) GEOS, and (F) GENS. RMSD of the RMalign’s solution (4.59 Å) was the threshold for the other algorithms. The target structure is colored green, the aligned fragment of the model is yellow, and the non-aligned one—magenta.

In the second set of experiments, we aligned examples of RNA structures using GEOS, GENS, and competitive algorithms, including those available only through webservers. As the first example, we chose structures from the third challenge of RNA-Puzzles (Cruz et al. 2012). In this puzzle, the predictors targeted the tertiary structure of a glycine riboswitch (PDB id: 3OWZ) (Huang et al. 2010) and submitted 12 *in silico* generated models of this molecule. We took model 2 from Das group (PZ3-Das-2; RMSD = 12.19 Å) to align it with the crystal structure of the target *T* (84 nts). We ran RMalign—the best according to quantitative analysis (cf. Fig. 1)—to align PZ3-Das-2 with *T* regardless of the sequence. It aligned 49 nucleotides of the model with an RMSD score 4.59 Å. This value served as a threshold for STAR3D, Rclick, GEOS, and GENS, which were also executed in sequence-independent mode. SETTER uses threshold values, but it turns out that they apply to the alignment of single nucleotides and not entire fragments. For this reason, this program was run with the default settings. Figure 2 shows the alignments obtained with the actual RMSD and the length listed aside.

**Figure 3.**
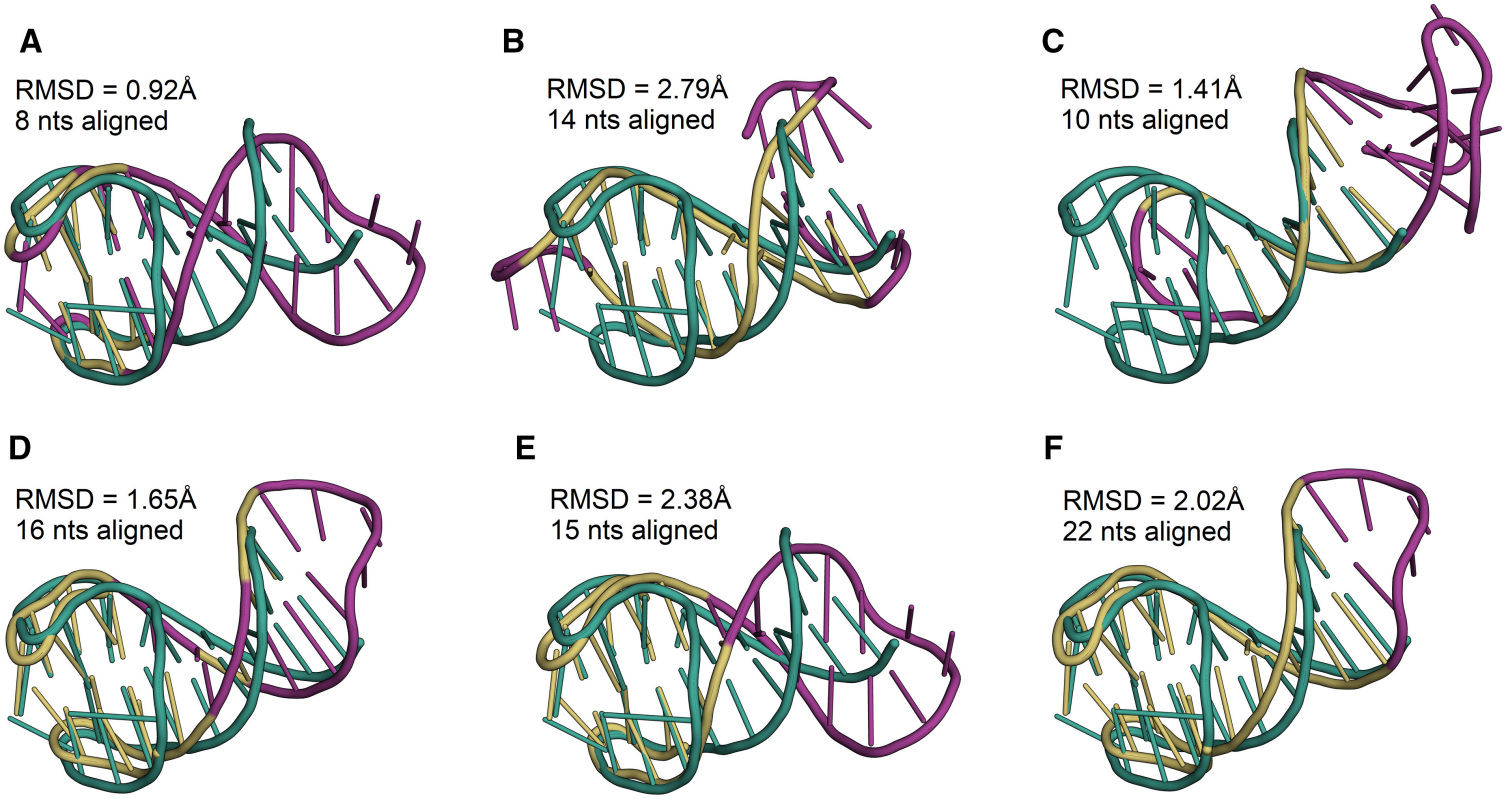
Two structures containing quadruplexes (PDB IDs: 6GN7, 2M8Z) aligned by (A) R3D Align, (B) SETTER, (C) STAR3D, (D) Rclick, (E) RMalign/RNA-align, and (F) GENS/GEOS. The 6GN7 structure is colored green, aligned fragments of 2M8Z are yellow, and non-aligned are magenta.

Next, we tested the algorithms on quadruplexes, specific, highly polymorphic structures found in nucleic acids. We searched the database of experimentally determined conformations of these motifs (Zok et al. 2022) and selected two instances with a similar secondary structure topology (Popenda et al. 2020). Both are hybrids of a duplex and a 2-tetrad unimolecular G-quadruplex. The first, a 26-mer DNA, comes from a complex with human alpha thrombin (PDB id: 6GN7) (Troisi et al. 2018) and entered the algorithms as a target. The second, a 27-mer DNA (PDB id: 2M8Z) (Lim and Phan 2013), was treated as a model; it was the one that would be transformed during alignment. We ran competing programs with default settings. RMalign and RNA-align provided exactly the same solution with actual RMSD = 2.38 Å. We set this value as the threshold for the GEOS and GENS algorithms. Both found alignment that included the same subset of nucleotides. Rclick, which requires a threshold value, was run with a threshold of 3 Å—the input value accepted by this algorithm must be in the range of 3–6 Å. SupeRNAlign did not return any result. Figure 3 shows all the solutions returned in the experiment.

**Figure 4.**
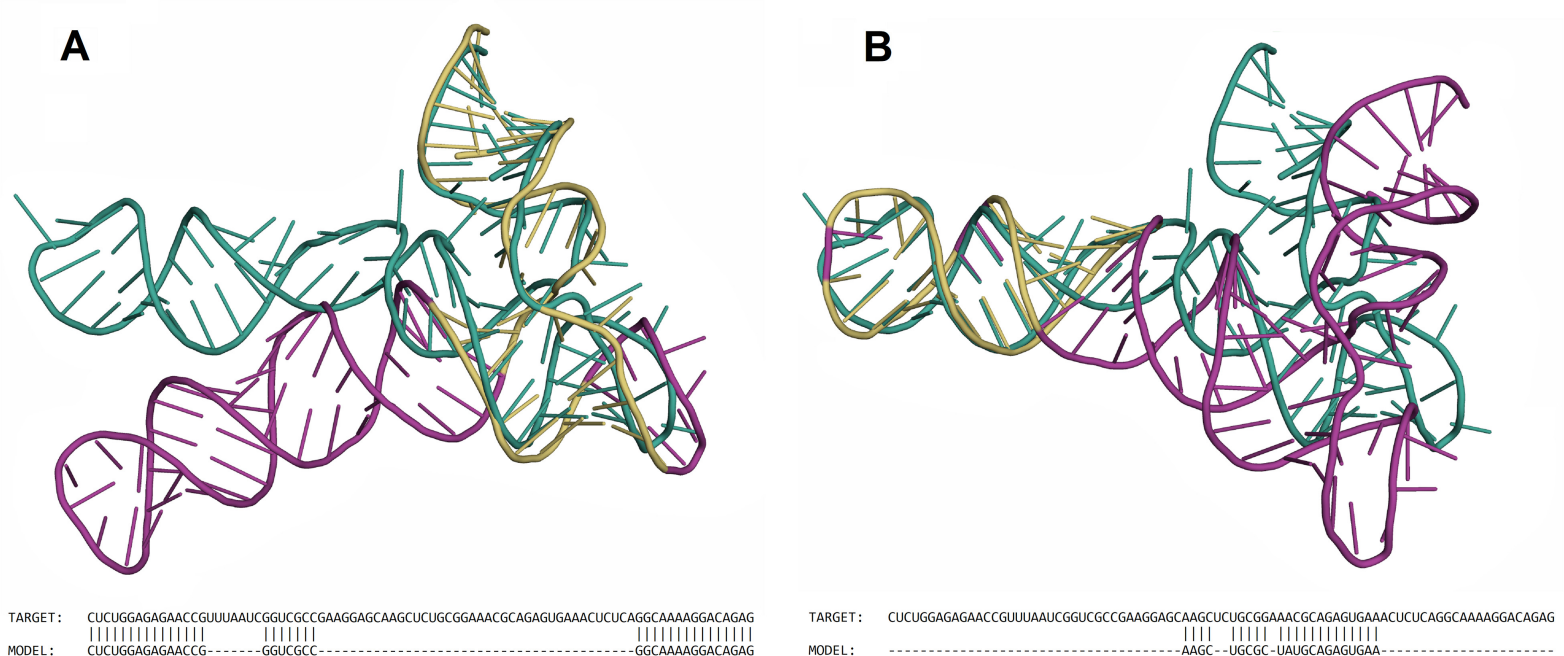
Different alignments between one of the predicted models and the target of Puzzle 3 found by GENS in sequence-dependent mode with threshold = 3.0 Å.

In the third experiment, we looked at different solutions generated by the GENS algorithm. Again, we took the target from challenge 3 of RNA-Puzzles and one of the models submitted there, namely PZ3-Chen-1. GENS was run in sequence-dependent mode with a threshold equal to 3 Å and found two disjoint solutions. In the first one (Fig. 4A), the aligned fragments of the PZ3-Chen-1 model have a total length of 37 nucleotides, representing 44% of the size of the whole molecule. They are located at the 3′ and 5′ ends of the chain. The actual RMSD of this alignment scores 2.96 Å. The alternative solution (Fig. 3B), located in the middle of the chain, is shorter, with 22 nucleotides (26% of the 84 nt-long structure) aligned with the corresponding fragment of the target. The actual RMSD of this solution is 2.62 Å. All fragments identified in these alignments show a high similarity to the target. However, we would not catch them easily when applying a global alignment approach. That is why GENS is helpful here—the greatest advantage of this algorithm is its ability to find all substructures aligning at a given threshold of the distance measure threshold, that is, all similar fragments in the compared structures.

### 3.3 Execution time and repeatability of the results

In these experiments, we focused exclusively on GEOS and GENS. First, we checked the repeatability of their results. Both algorithms are heuristics. Thus, they find suboptimal solutions that can vary between different runs for the same input data. In the experiment, we selected the structures of three targets from the benchmark set (targets in Puzzle 1, 3, and 4) and three models predicted per each targeted sequence (PZ1-Bujnicki-4, PZ1-Das-4, PZ1-Santalucia-4, PZ3-Chen-1, PZ3-Das-1, PZ3-Dokholyan-2, PZ4-Adamiak-4, PZ4-Bujnicki-1, PZ4-Mikolajczak-1). We searched for alignment for each model-target pair with the default RMSD threshold (3.5 Å). Each heuristic participated in 18 experiments—9 sequence-dependent alignments and 9 sequence-independent ones. Each experiment was repeated 125 times. We compared the results from all 125 runs for a given instance to compute the minimum and maximum lengths of alignment, the average, and the standard deviation. These results are presented in Supplementary Table S2. We found that GEOS was usually repeatable for the particular pair of 3D structures and the given values of the configuration parameters. Only in one case out of 18 did it find different alignments, with a small difference of 1 nucleotide. GENS, by design, generates multiple alternative alignments and can align various fragments of the structures. The experiment proved this property. In 14 experiments, GENS found various alignments. They varied in length by 2–38% (see also Supplementary Fig. S4).

Separately, we analyzed the execution times of GEOS and GENS as a function of the instance size. First, we computed the times for all instances in the set of RNA-Puzzles containing 22 target structures and 1006 RNA 3D models predicted *in silico*. Supplementary Fig. S5 estimates the trendline determined based on processing the RNA-Puzzles dataset. As the sizes of structures in this collection do not exceed 200 nts, we performed an additional experiment to test the efficiency of GEOS and GENS for larger RNAs. To collect the data for this experiment, we searched RNAsolo (Adamczyk et al. 2022) and BGSU RNA Hub (Leontis and Zirbel 2012), and we selected 8 relevant equivalence classes. Each of them contained homologous structures (with sizes between 100 and 700 nts), one of them being a representative of the class (see Supplementary Table S3). The algorithms looked for an alignment between the representative and other members of the same class. The results of this experiment are presented in Supplementary Fig. S6. For structures up to 200 nts, GEOS finishes computation before 60 s and GENS executes within a maximum of 35 s. For larger structures (>500 nts), GEOS still performs very well and finishes the computation before reaching the stop criterion. GENS is computationally too expensive for large structures and we do not recommend it for molecules above 300 nucleotides.

## 4 Conclusion

In this article, we address the flexible alignment of 3D RNA structures. The problem is computationally hard, as demonstrated for its protein version (Li 2013). This means that no exact algorithm can find its optimal solution in polynomial time. To date, several computational methods have been developed to solve this problem, SARA (Capriotti and Marti-Renom 2008), LaJolla (Bauer et al. 2009), R3D Align (Rahrig et al. 2010), iPARTS (Wang et al. 2010), SETTER (Hoksza and Svozil 2012), STAR3D (Ge and Zhang 2015), Rclick (Nguyen et al. 2017), SupeRNAlign (Piatkowski et al. 2017), RMalign (Zheng et al. 2019), and RNA-align (Gong et al. 2019). Most of them are still available.

We introduced two heuristics, that applied concurrency processing, to flexibly align 3D RNA structures, geometric search (GEOS), and genetic search (GENS). We compared them with existing methods that addressed the same problem. To ensure fairness of the comparison, we applied the actual RMSDs of the solutions obtained from competitive methods to constrain our algorithms and ranked all alignments according to their lengths. High-throughput tests on the RNA-Puzzles benchmark set showed that GEOS and GENS outperformed other methods on this criterion.

GEOS and GENS are available in the GitHub repository of the RNApolis group (Szachniuk 2019), ready to be used in future experiments and incorporated as components in various bioinformatics systems. Their uniqueness results from combining features dispersed among other methods, the most important of them being two modes of operation and a user-defined RMSD threshold. Provided within a standalone application, they facilitate finding alignments in multi-model datasets. However, aware of the demand for user-friendly bioinformatics tools, we plan to prepare a web server with GEOS and GENS working in the backend layer. Among other things, it will provide support for additional input parameters, visualization of results, and automatic processing of DNA structures.

## Supplementary Material

btad315_Supplementary_Data
