# Supplementary material for: High-quality, customizable heuristics for RNA 3D structure alignment
Source: Bioinformatics. 2023 May 11;39(5):btad315. doi: 10.1093/bioinformatics/btad315 (PMC10199316; doi:10.1093/bioinformatics/btad315)
Supplement: btad315_Supplementary_Data [file btad315_supplementary_data.pdf]

Supplementary Material

High-quality, customizable heuristics for RNA 3D structure alignment

Michal Zurkowski[1], Maciej Antczak[1,*], Marta Szachniuk[1,2,*]

[1] Institute of Computing Science, Poznan University of Technology, 60-965 Poznan, Poland

[2] Institute of Bioorganic Chemistry, Polish Academy of Sciences, 61-704 Poznan, Poland

*To whom correspondence should be addressed:

mantczak@cs.put.poznan.pl, mszachniuk@cs.put.poznan.pl

Figure S1. Flowchart of GEOmetric Search algorithm (GEOS): (A) identification of a kernel, (B) building the kernel-based alignment.

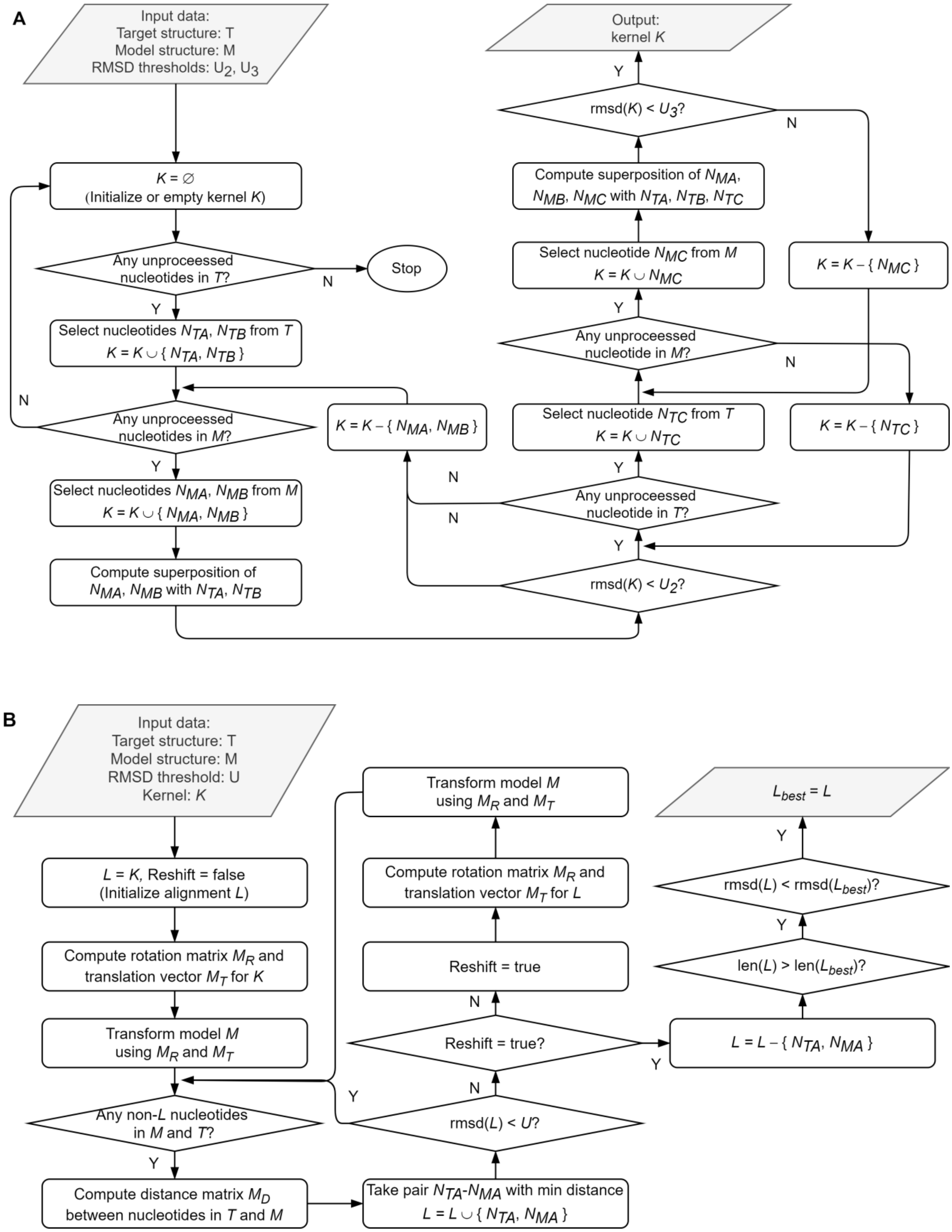

Figure S2. Schematic representation of mutations in the GENS algorithm.

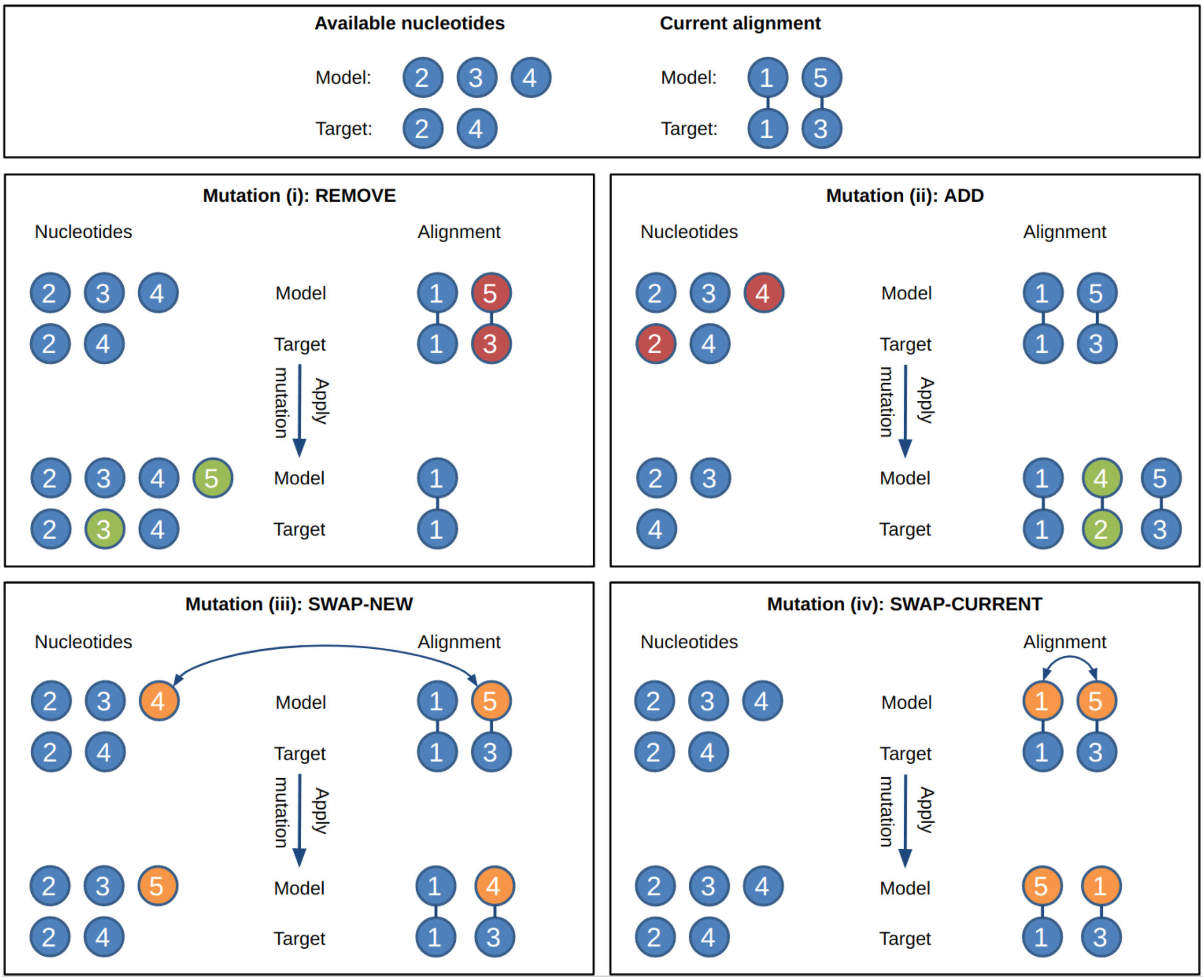

Figure S3. Average coverage of the target structure by aligned fragments of models submitted in a given puzzle. Alignments found by (A) R3D Align, (B) STAR3D, (C) SupeRNAlign, (D) RMalign, and (E) RNA-align.

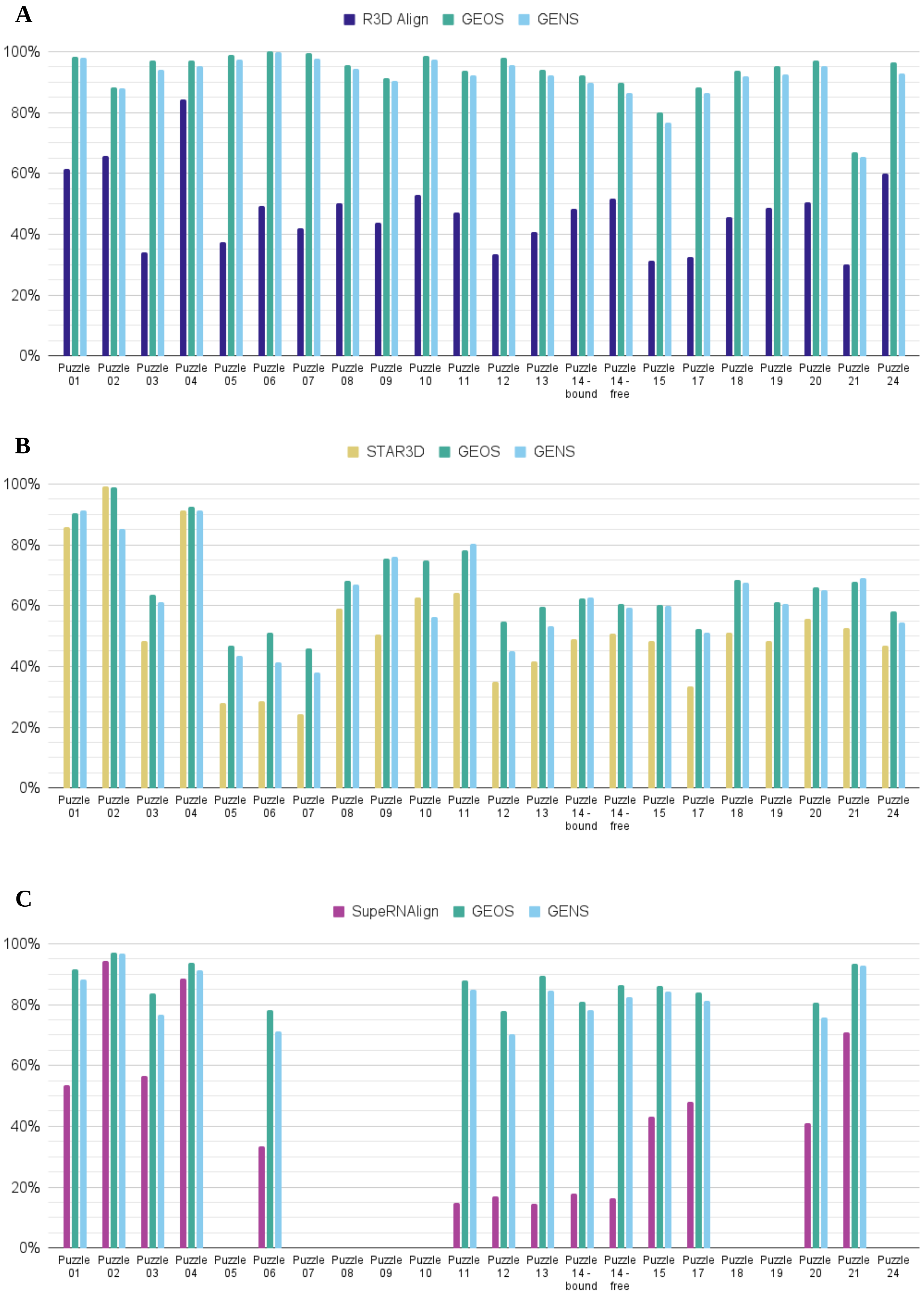

D

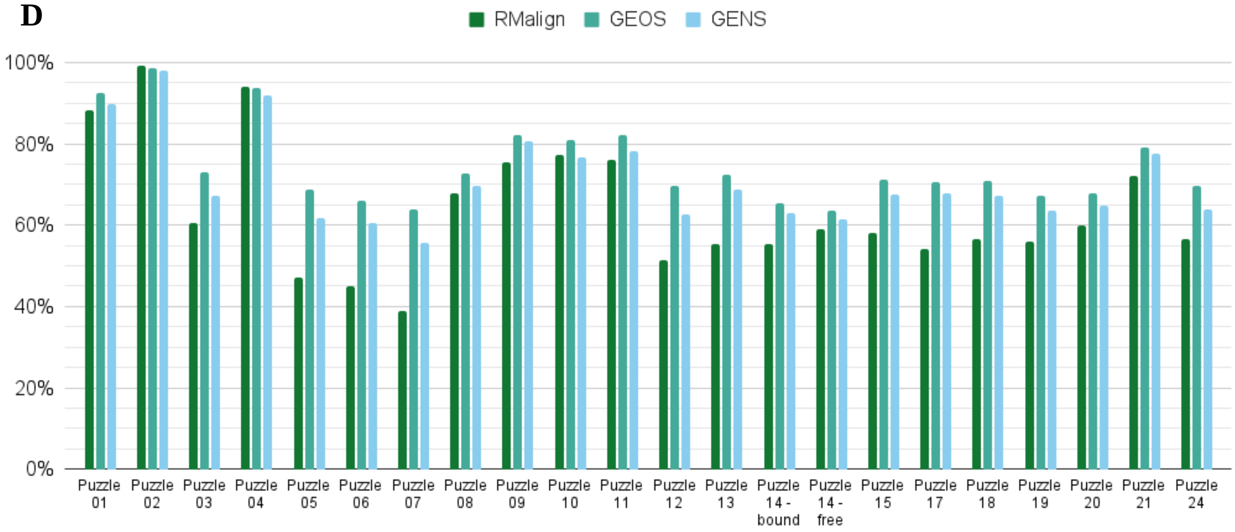
RMalign
GEOS
GENS
100%
80%
60%
40%
20%
0%
Puzzle 01
Puzzle 02
Puzzle 03
Puzzle 04
Puzzle 05
Puzzle 06
Puzzle 07
Puzzle 08
Puzzle 09
Puzzle 10
Puzzle 11
Puzzle 12
Puzzle 13
Puzzle 14 - bound
Puzzle 14 - free
Puzzle 15
Puzzle 17
Puzzle 18
Puzzle 19
Puzzle 20
Puzzle 21
Puzzle 24

E

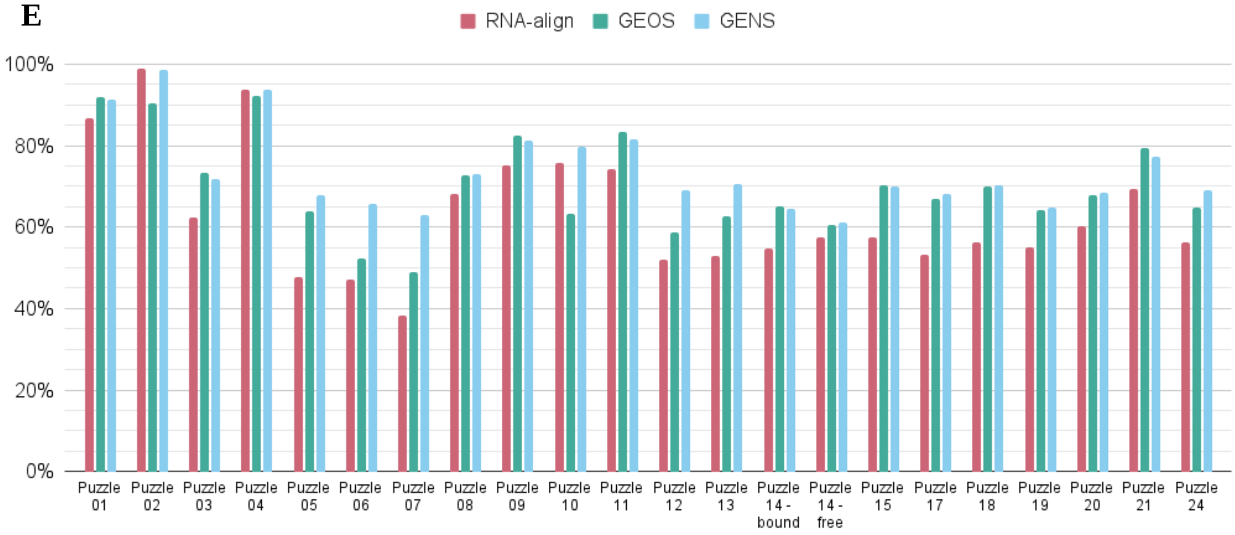
RNA-align
GEOS
GENS
100%
80%
60%
40%
20%
0%
Puzzle 01
Puzzle 02
Puzzle 03
Puzzle 04
Puzzle 05
Puzzle 06
Puzzle 07
Puzzle 08
Puzzle 09
Puzzle 10
Puzzle 11
Puzzle 12
Puzzle 13
Puzzle 14 - bound
Puzzle 14 - free
Puzzle 15
Puzzle 17
Puzzle 18
Puzzle 19
Puzzle 20
Puzzle 21
Puzzle 24

Figure S4. Distribution of the lengths of alignments found by GEOS in the experiment, in which the target structure of Puzzle 01, Puzzle 03, and Puzzle 04 were aligned with three models predicted in these puzzles. GENS was run with the default RMSD threshold (3.5Å) and performed 125 times for each instance.

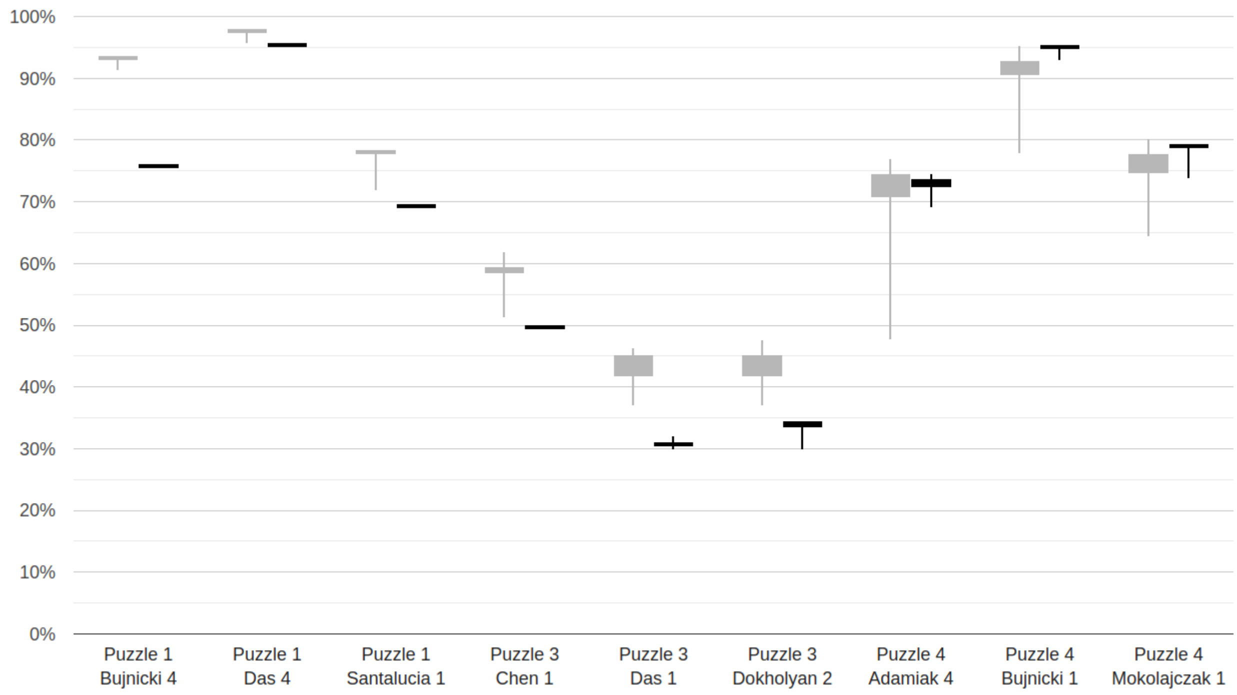

Figure S5. Trendline for execution time of GEOS (A) and GENS (B) computed when aligning small and medium-size structures from the RNA-Puzzles dataset.

A

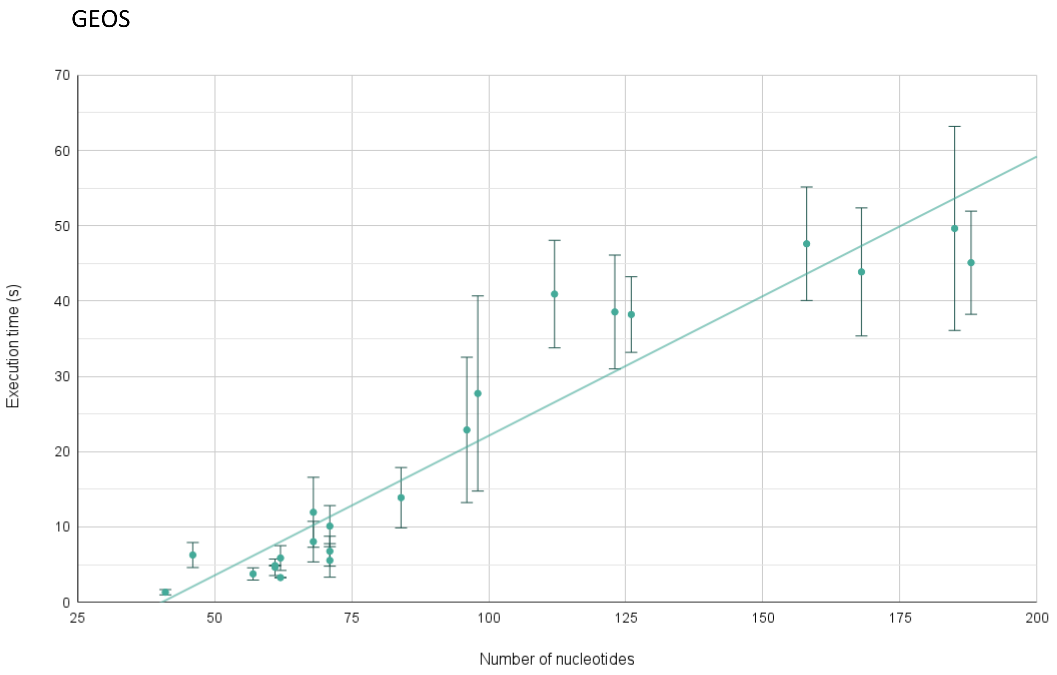

B

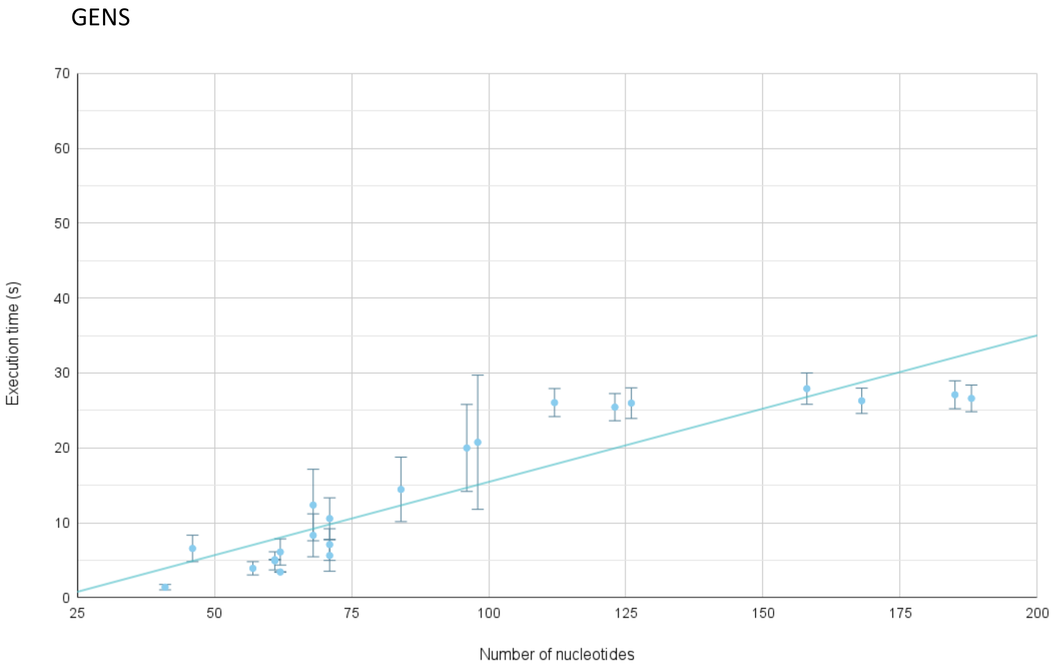

Figure S6. Trendline for execution time of GEOS (A) and GENS (B) computed when aligning large structures from the RNAsolo/BGSU dataset.

A GEOS

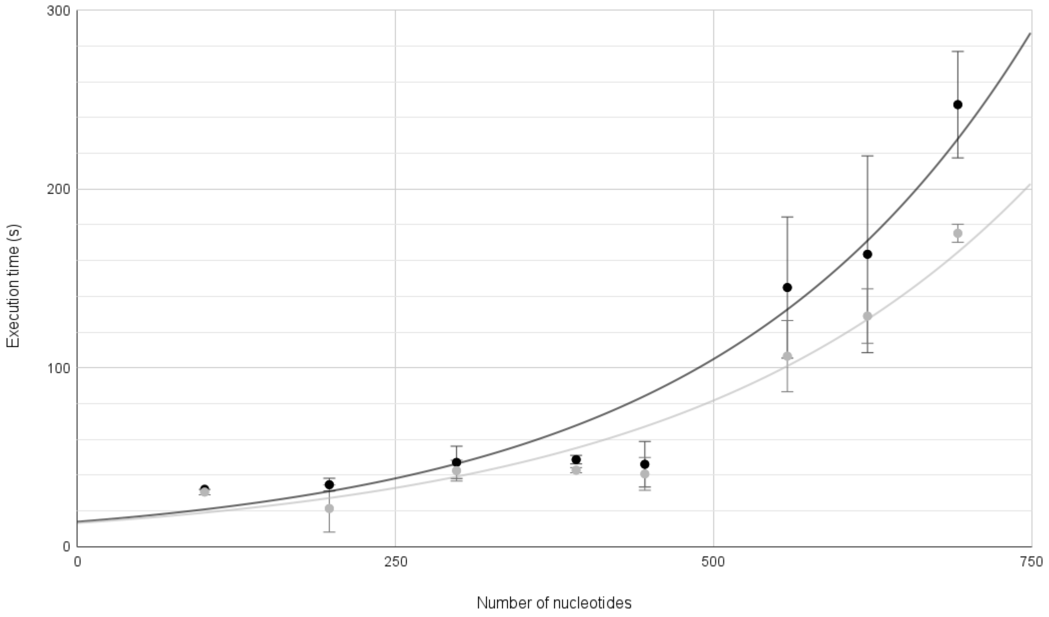

B GENS

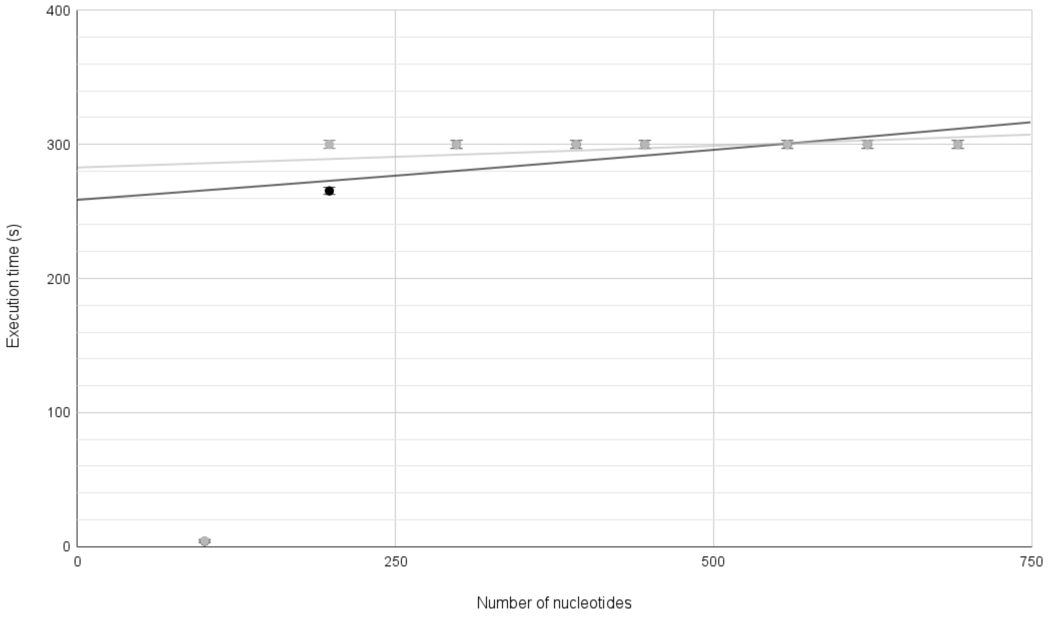

Table S1. The number of duels won and lost by each algorithm.

	GEOS	GENS	R3D Align	# Duels won
GEOS	–	580	1014	1594
GENS	16	–	1010	1026
R3D Align	2	4	–	6
# Duels lost	18	584	2024	–
	GEOS	GENS	STAR3D	# Duels won
GEOS	–	547	947	1494
GENS	200	–	915	1115
STAR3D	30	72	–	102
# Duels lost	230	619	1862	–
	GEOS	GENS	SupeRNAlign	# Duels won
GEOS	–	201	262	463
GENS	1	–	260	261
SupeRNAlign	0	1	–	1
# Duels lost	1	202	522	–
	GEOS	GENS	RMalign	# Duels won
GEOS	–	800	854	1654
GENS	1	–	759	760
RMalign	78	181	–	259
# Duels lost	79	981	1613	–
	GEOS	GENS	RNA-align	# Duels won
GEOS	–	551	872	1423
GENS	262	–	849	1111
RNA-align	68	201	–	269
# Duels lost	330	752	1718	–

Table S2. Lengths of alignments found by GEOS and GENS in three rounds of experiment. Three predicted models were aligned with the target structure of (i) Puzzle 01 (46nts), (ii) Puzzle 03 (84nts), and (iii) Puzzle 04 (126nts). Both algorithms were run with the default RMSD threshold (3.5Å) and performed 125 times for each instance.

Round (i)		GEOS (seq-ind)	GEOS (seq-dep)	GENS (seq-ind)	GENS (seq-dep)
PZ1-Bujnicki-4 vs target	Min length	44	35	42	35
	Max length	44	35	43	35
	Avg length	44.0	35.0	42.9	35.0
	St. dev.	0.0	0.0	0.2	0.0
PZ1-Das-4 vs target	Min length	45	43	44	44
	Max length	45	43	45	44
	Avg length	45.0	43.0	44.8	44.0
	St. dev.	0.0	0.0	0.4	0.0
PZ1-Santalucia- 4 vs target	Min length	36	32	33	32
	Max length	36	32	36	32
	Avg length	36.0	32.0	35.6	32.0
	St. dev.	0.0	0.0	0.9	0.0
Round (ii)					
PZ3-Chen-1 vs target	Min length	53	41	43	42
	Max length	53	42	52	42
	Avg length	53.0	41.9	49.4	42.0
	St. dev.	0.0	0.2	1.6	0.0
PZ3-Das-1 vs target	Min length	38	26	31	25
	Max length	38	26	39	27
	Avg length	38.0	26.0	36.2	25.9
	St. dev.	0.0	0.0	1.9	0.6
PZ3-Dokholyan-2 vs target	Min length	37	29	31	25
	Max length	37	29	40	29
	Avg length	37.0	29.0	36.4	28.3
	St. dev.	0.0	0.0	1.9	0.7
Round (iii)					
PZ4-Adamiak-4 vs target	Min length	97	94	60	87
	Max length	97	94	97	94
	Avg length	97.0	94.00	90.5	91.7
	St. dev.	0.0	0.000	5.5	1.3
PZ4-Bujnicki-1 vs target	Min length	119	120	98	117
	Max length	119	120	120	120
	Avg length	119.0	120.0	115.2	119.7
	St. dev.	0.0	0.0	2.7	0.7
Pz4-Mikolajczak-1 vs target	Min length	101	100	81	93
	Max length	101	100	101	100
	Avg length	101.0	100.0	95.6	98.9
	St. dev.	0.0	0.0	3.4	0.9

Table S3. Dataset used to test execution times of GEOS and GENS applied to find alignment between large RNA structures (100-700nts).

Subset	1	2	3	4	5	6	7	8
Representative	6DME_A	5JUP_EC	7B9V_2+6	7EZ2_N	6ZQc_D2	6N7R_R	6HIW_CA	8H2H_A
Size [nts]	100	198	298	392	446	558	621	692
Class id	86427.1	99969.1	1315.2	60848.2	62396.2	34961.3	60828.6	56999.3
# members	5	7	7	11	3	6	5	4
